# 10‐kHz Spinal Cord Stimulation for Chronic Postsurgical Pain: Results From a 12‐Month Prospective, Multicenter Study

**DOI:** 10.1111/papr.12929

**Published:** 2020-07-23

**Authors:** Mayank Gupta, James Scowcroft, Daniel Kloster, Maged Guirguis, Jonathan Carlson, Tory McJunkin, Gassan Chaiban, Atef Israel, Jeyakumar Subbaroyan

**Affiliations:** ^1^ Kansas Pain Management Overland Park Kansas U.S.A.; ^2^ KC Pain Centers Lee’s Summit Missouri U.S.A.; ^3^ Menorah Medical Center Overland Park Kansas U.S.A.; ^4^ Ochsner Health System New Orleans Louisiana U.S.A.; ^5^ AZ Pain Specialists Scottsdale Arizona U.S.A; ^6^ Nevro Corp Redwood City California U.S.A.

**Keywords:** 10‐kHz SCS, chronic postsurgical pain, visual analog scale

## Abstract

**Background:**

Chronic postsurgical pain (CPSP) can be caused by peripheral nerve injury (PNI) resulting from surgical procedures and has a significant neuropathic component. This prospective, single‐arm study was conducted to document the effectiveness of 10‐kHz spinal cord stimulation (10‐kHz SCS) as a treatment for patients with CPSP.

**Methods:**

Subjects with CPSP who were refractory to conventional medical interventions and reported pain scores of ≥5 cm on a 10‐cm VAS underwent trial stimulations lasting up to 14 days. Epidural leads were implanted at locations appropriate for the primary area of pain, and trials resulting in ≥40% pain relief were considered successful. Subjects with successful trials underwent implantation with a permanent 10‐kHz SCS system and were followed for 12 months after implantation.

**Results:**

Of the 34 subjects who underwent trial stimulation, 1 was withdrawn early and 29 (87.9%) had a successful trial and received a permanent implant. After 12 months of treatment, the mean VAS score decreased by 6.5 cm, the response rate was 88.0% (22/25), and 18 subjects (62.1%) were remitters with VAS scores sustained at ≤3.0 cm. Scores for all components of the short‐form McGill Pain Questionnaire 2 were significantly reduced, including affective descriptors of pain. Pain catastrophizing and vigilance, patient function, physical and mental well‐being, and sleep quality all improved over the course of the study. No neurologic deficits reported in the study.

**Conclusions:**

10‐kHz SCS is effective and tolerated in patients with CPSP, and further study of its clinical application in this population is warranted.

## Introduction

Chronic postsurgical neuropathic pain (CPSP) is a chronic pain syndrome recognized by the International Association for the Study of Pain (IASP) and the World Health Organization (WHO). CPSP is a diagnosis of exclusion defined as pain developing after surgery or tissue injury (including burns) and persisting for at least 3 months after the trauma occurred.^1^ Surgery or trauma preceded the pain in 41% of patients in a chronic pain clinic,^2^ and the estimated incidence of CPSP varies widely after surgery, from 6% following cesarean section to ≥50% following amputation or coronary artery bypass surgery.^3^, ^4^ In most cases, there is an iatrogenic peripheral nerve injury (PNI) associated with CPSP.

Neuropathic CPSP has been associated with more intense, persistent, and impactful pain^4^ and is most often observed following thoracic surgery (66% of patients with CPSP) and breast surgery (67%), while it is rare after total hip or knee arthroplasty (6% each).^5^ First‐line pharmacologic treatments include calcium channel antagonists, antidepressants, topical lidocaine, and topical capsaicin, as well as acetaminophen, nonsteroidal anti‐inflammatory drugs, and weak opioids. Second‐line treatments include neuromodulation, pulsed radiofrequency, nerve blocks, and nerve ablation.

Pulsed radiofrequency treatment has reduced pain in postsurgical settings, but studies showing long‐term efficacy are lacking.^6^ Peripheral nerve stimulation (PNS) can be effective for treating neuropathic pain^7^ but requires implantation of electrodes near the nerve to be stimulated, making wearing and charging the device more challenging for patients with pain. The risk for lead migration and the need for lead stabilization is also higher in the periphery compared to conventional spinal cord stimulation (SCS).

Studies evaluating paresthesia‐based SCS in CPSP have reported effective pain relief in some cases. Preclinical studies have demonstrated that low‐frequency SCS can modulate genes upregulated after PNI in an amplitude‐dependent fashion, increases paw withdrawal thresholds, and suppresses allodynia, explaining the potential mechanism of analgesia in this pain condition.^8^, ^9^, ^10^ An early study of patients with pain from multiple etiologies in the CPSP group of chronic pain disorders, including reflex sympathetic dystrophy and causalgia, spinal lesions due to trauma, and failed back surgery, found that nearly 89% experienced reduced pain during trial SCS stimulation, but after 3 years, this decreased to 28%.^11^ A small study of chronic, intractable groin and low back pain following herniorrhaphy reported pain relief of ≥75% in all patients after 1 year of SCS treatment.^12^

Despite the unmet need for effective nonpharmacologic treatments for CPSP, published data regarding this pain condition are sparse. Many published studies of patients with CPSP only included those with complex regional pain syndrome, and the larger population with CPSP has not been examined in detail. Therefore, this study was conducted to evaluate 10‐kHz SCS for the treatment of likely neuropathic CPSP and included neurological assessments before and after stimulation. Unlike other neuromodulation methods tested, 10‐kHz SCS is paresthesia independent, which avoids the burdens associated with paresthesia mapping and the discomfort associated with this sensation.

## Methods

### Study Design and Subjects

**Figure 1:**
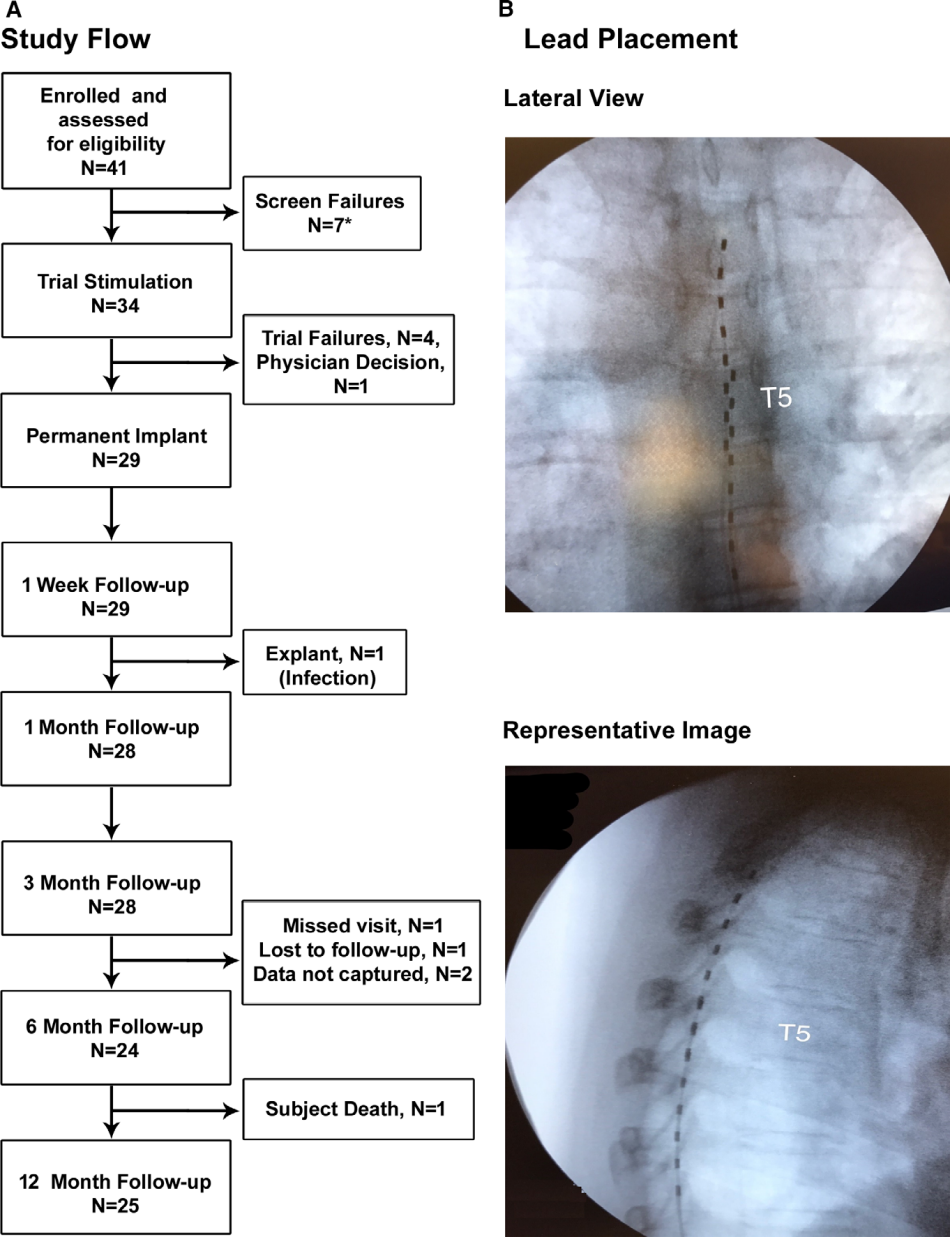
A, CONSORT diagram of patient flow through the study. B, X‐ray images of octapolar lead placement in epidural space. *Reasons for screen fail: withdrew consent (#3), withdrawn by physician due to psychiatric issues (#3), cognitive ability to handle programmer/charger (#1).

This was a single‐arm, prospective, multicenter, post‐market study. The Western Investigational Review Board reviewed and approved the investigational plan, amendments, and informed consent forms before implementation, and the study complied fully with the U.S. Code of Federal Regulations and recommendations guiding physicians in biomedical research by the 18th World Medical Assembly, Helsinki, Finland. The study design and patient flow are shown in Figure S1 and Figure 1A.

Eligible subjects were affiliated with, or referred to, the clinical investigation sites and were diagnosed with chronic, focal, neuropathic pain following surgery involving the trunk or the limbs limited to 1 to 2 dermatomes with an average intensity of ≥5 cm on a 10‐cm VAS over 7 days. All subjects had scores of ≥4 on the Douleur Neuropathique 4 (DN4) neuropathic pain diagnostic questionnaire, a threshold that captures patients with a ≥70% chance of having neuropathic pain.^13^ Full inclusion criteria are listed in Table S1. Patients with chronic pain from failed back surgery, a progressive neurological disease, or previous experience with SCS therapy were excluded. Full exclusion criteria are listed in Table S2.

### Procedures

All eligible enrolled subjects underwent trial stimulations lasting up to 2 weeks. Investigators placed octapolar leads in the epidural space based on each subject’s pain location (Figure 1B). The leads spanned from C2 to C6 vertebral levels in subjects with upper limb pain and from T8 to T12 in patients with lower limb pain. In subjects with trunk pain, the leads were positioned such that the most proximal contact was ≥2 dermatomes superior to the most rostral dermatomal distribution of pain. The devices for all subjects were programmed to perform simple bipole searches with starting amplitudes and step sizes determined by lead location, which were divided into cervical, upper thoracic, and lower thoracic categories.

Trial stimulation resulting in at least 40% pain relief were deemed successful, and these subjects underwent implantation with a permanent 10‐kHz SCS system (Senza System; Nevro Corp., Redwood City, CA, U.S.A.). A threshold of 40% pain relief exceeds the minimum clinically meaningful improvement for VAS scores (≥30% pain relief)^14^ but is less stringent than the 50% threshold typically used to define responses in patients with permanently implanted systems.^15^, ^16^, ^17^ The stimulation parameters were a frequency of 10 kHz and a pulse width of 30 microseconds, and investigators individually adjusted pulse amplitudes for each subject to maximize pain relief without paresthesia.

### Outcomes Assessments

Outcomes were assessed before beginning the trial stimulation (baseline) and at follow‐up visits. The primary outcome, pain intensity, was assessed using VAS scores. In contrast to the trial stimulation, subjects with permanent implants were classified as responders if their VAS scores declined by 50% or more, a commonly used threshold.^15^, ^16^, ^17^ Subjects were also classified as remitters or non‐remitters based on a threshold VAS score of 3.0 or less for at least 6 months, a threshold based on a post hoc analysis of the SENZA‐RCT study.^18^

Pain characteristics were evaluated using the short‐form McGill Pain Questionnaire 2 (SF‐MPQ‐2).^19^ The subjects’ tendency to catastrophize over their pain was determined using the Pain Catastrophizing Scale (PCS),^20^ and attention to pain was quantified using the Pain Vigilance and Awareness Questionnaire (PVAQ).^21^ The subjects’ perceptions of treatment were assessed by surveying subject satisfaction and using the patient‐reported Global Impression of Change scale (PGIC), and the clinicians’ perceptions of the study treatment were assessed using the clinician‐reported Global Assessment of Change scale (CGIC).^22^ The Pain Disability Index (PDI)^23^ quantified functional impairment, and the Global Assessment of Functioning scale (GAF)^24^ quantified subjects’ overall mental health. Finally, investigators used the 12‐Item Short‐Form Health Survey (SF‐12)^25^ to assess subjects’ overall well‐being and the 3‐item Pain and Sleep Questionnaire (PSQ‐3) Index^26^ to determine sleep quality.

Routine standard of care neuro‐assessments were performed at baseline, at the end of the trial stimulation, and after 3 and 12 months of stimulation. Results were documented as “maintenance,” “improvement,” or “deficit” for sensory, motor, and reflexes. To maintain consistency, the same investigator conducted the neuro‐assessments at all time points.

### Statistics

All outcomes were analyzed using descriptive statistics. Categorical variables were reported as counts and percentages, and continuous variables were reported as means ± standard error of the mean (SEM) intervals, as appropriate. To determine the statistical significance of longitudinal results, baseline measures were compared to follow‐up values using 2‐tailed paired *t*‐tests with a fixed variable (post‐implantation measurement time) and a random variable (subject). The threshold for statistical significance was *P* ≤ 0.05. All statistical analysis was performed using Microsoft Excel 2016 (Microsoft Corporation, Redmond, WA, U.S.A.). Safety results were reported in the intent‐to‐treat population (all subjects who underwent a trial stimulation), and efficacy results were reported in the per‐protocol population (subjects who received an implant and completed 3‐month follow‐up).

## Results

### Stimulation Trials

In all, 34 study subjects, including 21 women (61.8%), enrolled in this study and underwent trial stimulation, and their demographic information is summarized in Table 1. The mean age was 54.4 ± 2.1 years, with a range from 30.5 to 85.5 years, and the mean time since diagnosis was 5.0 ± 1.0 years. The subjects had a variety of surgeries (Table S3), and 23 (67.6%) had lower limb pain, 9 (26.5%) had trunk pain, and 2 (5.9%) had upper limb pain. Trial stimulation was successful in 29 of 33 subjects (87.9%), while 1 subject was withdrawn during the trial stimulation due to physician decision. Of the 29 subjects who received permanently implanted SCS systems, 25 completed the entire study and attended the 12‐month follow‐up visit. A total of 4 subjects were withdrawn from the study after permanent implantation, including 2 who had the device explanted due to pain at the implantable pulse generator site in one case and infection in the other (Figure 1), and a third subject who was lost to follow‐up. There was also a single death during the study (suicide), and this event was deemed unrelated to the study treatment.

**Table 1:** Demographics and Clinical Characteristics

Characteristics	Subjects (*N *= 34)
Gender, *n* (%)	
Female	21 (61.8)
Male	13 (38.2)
Age at enrollment (years)	
Mean ± SD	54.4 ± 12.4
Range	30.5 to 85.5
Years since diagnosis	
Mean ± SD	5.0 ± 6.1
Range	0.0 to 29.0
Ethnicity, *n* (%)	
Hispanic/Latino	3 (8.8)
Non‐Hispanic/Latino	31 (91.2)
Race, *n* (%)	
Black/African American	4 (11.8)
Mexican	1 (2.9)
White	29 (85.3)
Pain location, *n* (%)	
Lower limb	23 (67.6)
Trunk	9 (26.5)
Upper limb	2 (5.9)
Pain symmetry, *n* (%)	
Bilateral	6 (17.6)
Midline	2 (5.9)
Unilateral	26 (76.5)
Pain present prior to surgery? *n* (%)	
Yes	18 (52.9)
Baseline VAS score (cm)	
Mean ± SEM	7.9 ± 1.3
Range	5.2 to 10.0

SD, standard deviation.

### Safety

A total of 6 study‐related adverse events (AEs) were recorded during the study, and 6 subjects were affected (17.6%; *n* = mild, *n* = 3 moderate). These AEs primarily involved the implantation procedure or the device itself. A total of 15 serious adverse events (SAEs) were observed in 9 subjects (26.5%) during the study. All but one of them were unrelated to the study, device, or the procedure. The single procedure‐related SAE (implant site infection) was resolved when the device was explanted. Neuro‐assessments revealed no deficits in neural functioning after the trial stimulation, nor after 3 months or 12 months of 10‐kHz SCS treatment. Interestingly, 9 of 28 subjects (32.1%) and 9 of 24 subjects (37.5%) demonstrated improvements in neuro‐assessments at the 3‐month and 12‐month follow‐up visits, respectively.

### Pain Outcomes

**Figure 2:**
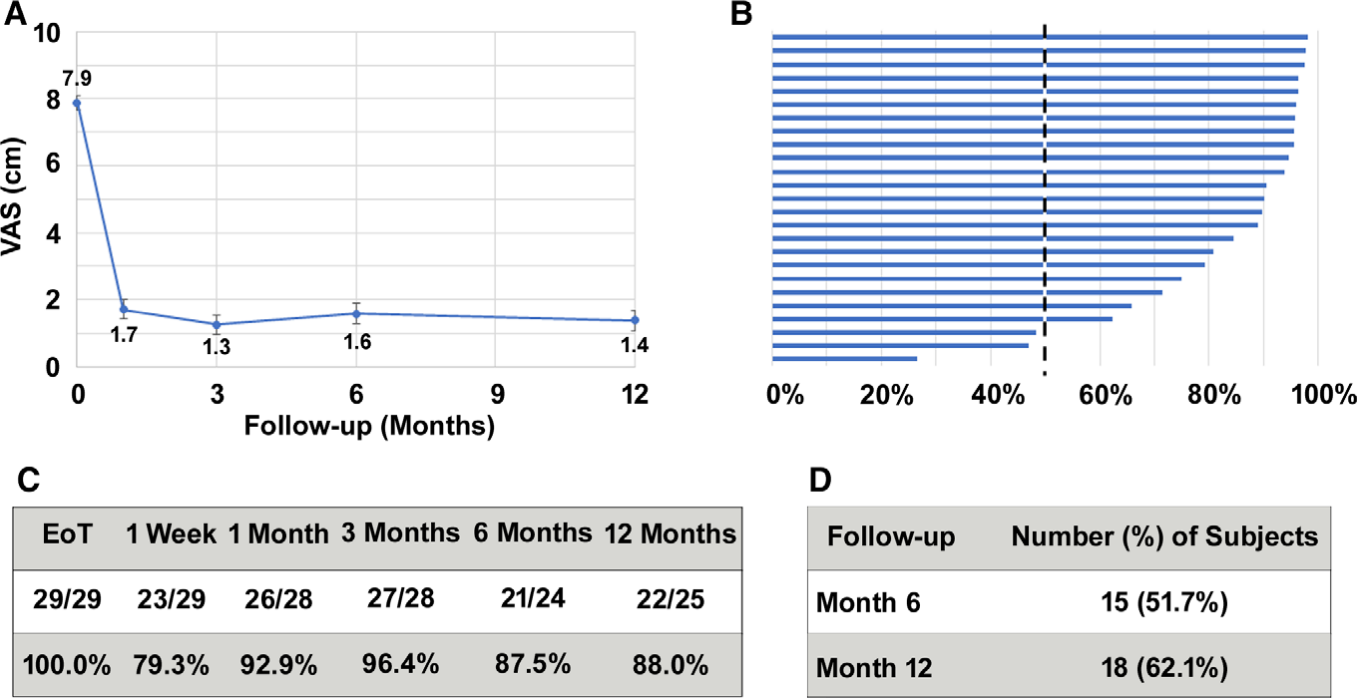
Pain intensity in patients with chronic postsurgical pain decreased after treatment with 10‐kHz spinal cord stimulation. A, VAS scores at baseline and follow‐up visits (mean ± SEM). B, Tornado plot showing percentage of pain relief for individual subjects at the 12‐month time point. C, Responder rate (≥50% pain relief) after the trial stimulation and at follow‐up visits. D, Remitter rate (≤3.0 cm on the VAS) at 6‐ and 12‐month follow‐up visits. EoT, End of Trial.

**Figure 3:**
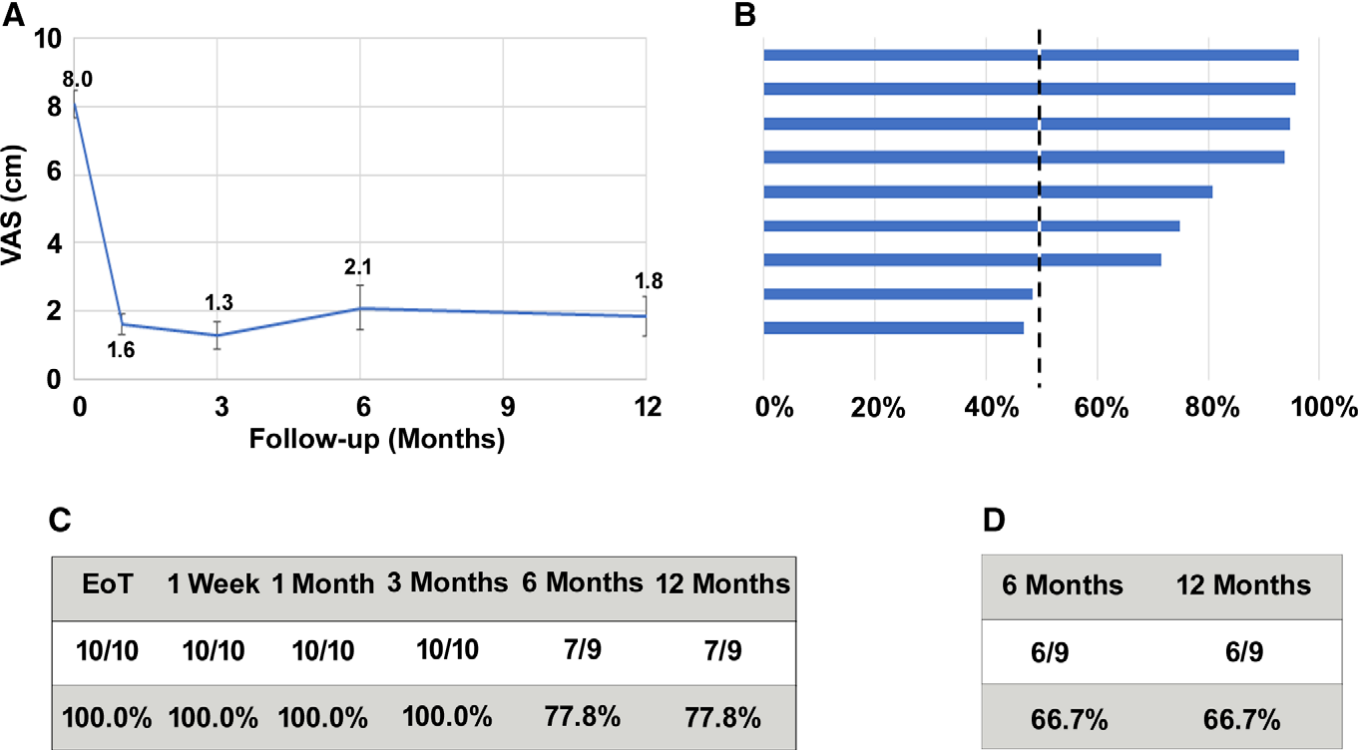
Pain intensity in patients with chronic postsurgical pain after knee replacement decreased after treatment with 10‐kHz spinal cord stimulation. A, VAS scores at baseline and follow‐up visits (mean ± SEM). B, Tornado plot showing percentage pain relief for individual subjects at the 12‐month time point. C, Responder rate (≥50% pain relief) after the trial stimulation and at follow‐up visits. D, Remitter rate (≤3.0 cm on the VAS) at 6‐ and 12‐month follow‐up visits. EoT, End of Trial.

Among the 29 subjects who had a successful 10‐kHz SCS trial, VAS scores declined by 6.2 cm after 3 months and 6.5 cm after 12 months of stimulation(Figure 2A). Study subjects with ≥50% pain relief in VAS score were defined as responders, as shown in Figure 2B, and the responder rate was 96.4% (27 of 28) after 3 months and remained consistently high after 12 months of treatment (22 of 25; 88.0%). After 1 year of treatment, 18 subjects (62.1%) were deemed remitters.^18^ A subgroup analysis conducted on subjects with lower limb pain following knee replacement surgery produced results similar to those found for the entire cohort (Figure 3). Mean VAS scores in the knee patients decreased from 8.0 cm at baseline to 1.6 cm after trial stimulation and remained low for 12 months of treatment. Responder rates were 77.8% (7 of 9) at 6 and 12 months of treatment, while 6 of 9 subjects were remitters (66.7%) at 12 months.

**Figure 4:**
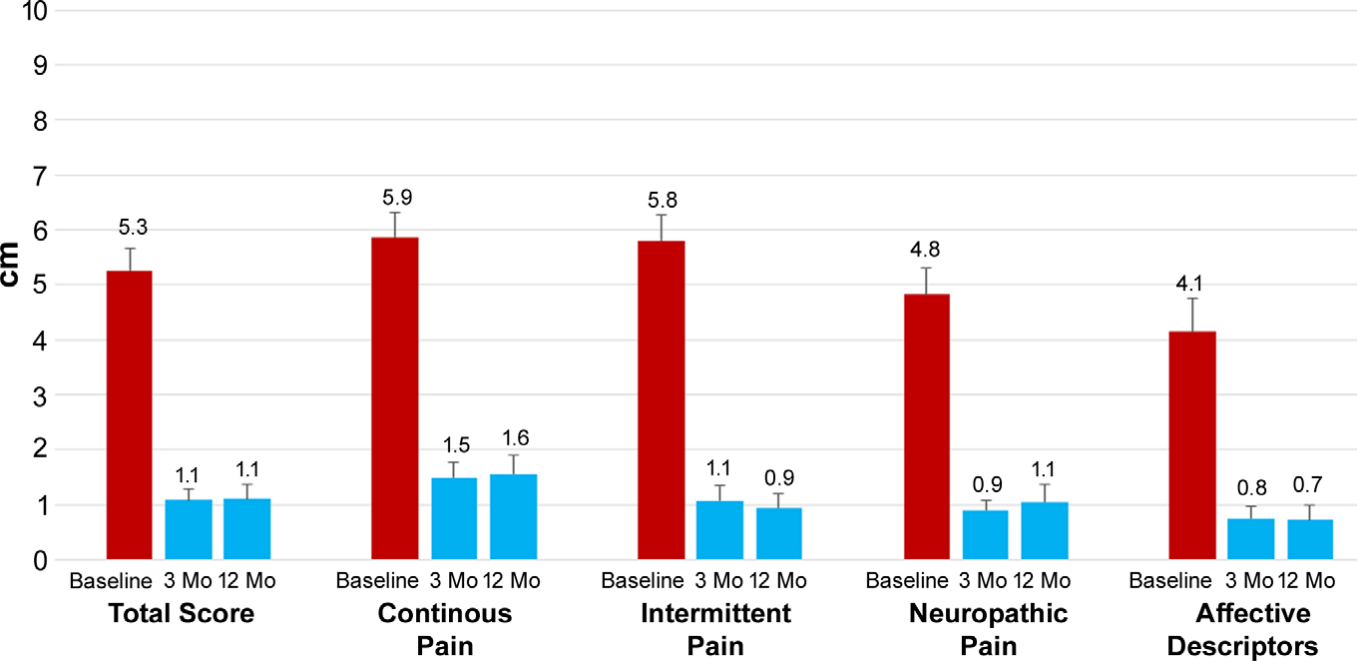
All subscales of pain were significantly reduced after treatment with 10‐kHz spinal cord stimulation. Scores from the short‐form McGill Pain Questionnaire 2 are shown at baseline and 3 and 12 months after treatment initiation for the total score and continuous pain, intermittent pain, neuropathic pain, and affective descriptor subscales.

Pain intensity was also assessed using the SF‐MPQ‐2, and the results are displayed in Figure 4. The total scores declined 4.2 points at both the 3‐month and 12‐month follow‐up visits, which was statistically significant (*P* < 0.001). After 12 months, the mean scores for neuropathic pain and affective descriptors of pain were 3.7 points and 3.4 lower than at baseline, respectively (*P* < 0.001 for both comparisons).

### Patient Perception of Pain

**Figure 5:**
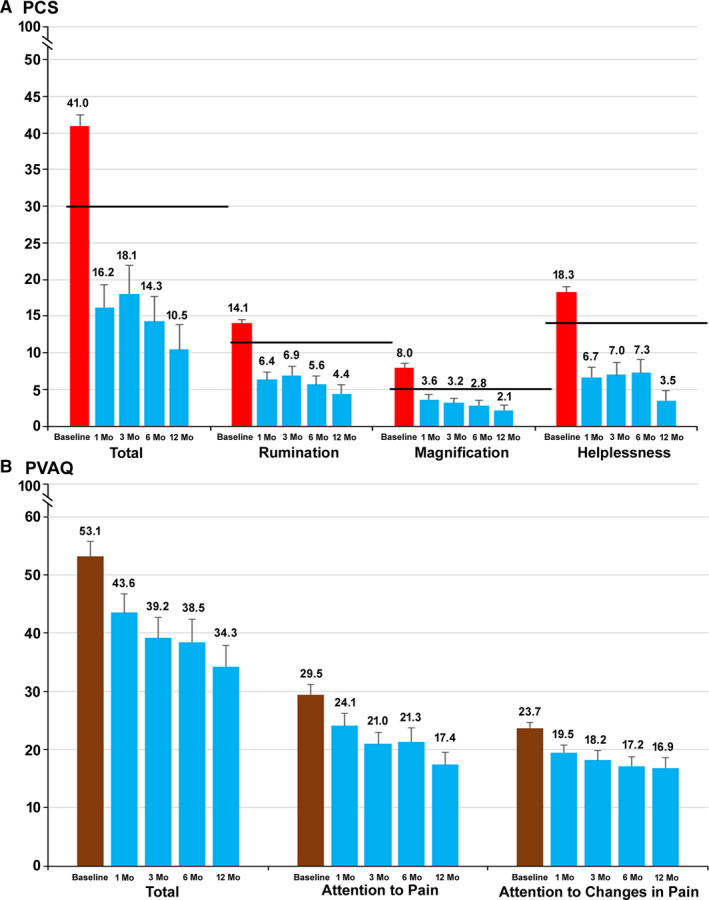
Subjects’ perceptions of pain decreased after treatment with 10‐kHz spinal cord stimulation. A, Mean total and subscale Pain Catastrophizing Scale (PCS) scores at baseline and 1, 3, 6, and 12 months after the start of treatment. The 75th percentile of the distribution is indicated for each score by the heavy black line. Threshold values are 30 (total), 11 (rumination), 5 (magnification), and 13 (helplessness). B, Mean total Pain Vigilance and Awareness Questionnaire (PVAQ) and subscale scores at baseline and 1, 3, 6, and 12 months after the start of treatment.

Subjects’ perceptions of pain were assessed using the PCS and the PVAQ, and the results are summarized in Figure 5. In the subjects who presented with clinically relevant catastrophization (*n* = 15, PCS total score ≥ 30), PCS scores decreased from a mean of 41.0 at baseline by 22.9 points after 3 months (*P* < 0.001) and 30.5 points after 12 months of stimulation (*P* < 0.001) (Figure 5A). By end of trial, nearly 50% of the subjects had improved to a subclinical level of catastrophization, and by 6 months postimplantation, none of the subjects presented a clinical level of catastrophization. This decrease was also observed in all 3 subscales of the PCS. At the 12‐month visit, the mean rumination score decreased by 9.7 points (*n* = 14, *P* < 0.001), the magnification score decreased by 5.9 points (*n* = 15, *P* < 0.001), and the mean helplessness score decreased by 14.8 points (*n* = 12, *P* < 0.001). The mean PVAQ scores of all subjects, as well as the mean scores of both the Attention to Pain and Attention to Changes in Pain subscales, decreased from baseline throughout the course of the study (Figure 5B). The mean total PVAQ score was 53.1 at baseline and decreased by 18.8 points at the 12‐month visit (*P* < 0.001).

The study subjects were surveyed about their satisfaction level with 10‐kHz SCS therapy. After 3 months, 27 of 28 subjects (96.4%) were satisfied or very satisfied with their treatment, and 23 of 25 subjects (92.0%) were satisfied or very satisfied after 12 months.

### Disability, Quality of Life, and Sleep

The patients’ and clinicians’ perceptions of 10‐kHz SCS treatment was assessed using the PGIC and CGIC. At 12 months postimplantation, 88.0% (22 of 25) of the subjects were reported to be “better” or “a great deal better” as assessed with both the PGIC and CGIC.

Multiple tools, including the GAF, PDI, and SF‐12, were used to assess subject quality of life, disability, and well‐being, respectively. The mean GAF score increased 17.9 points (24.4%) after 12 months of treatment, and the PDI score declined from 42.8 ± 2.3 at baseline to 13.8 ± 2.6 (*P* < 0.001). After 12 months, PDI scores exceeded the minimal clinically important difference of 8.5 to 9.5^27^ in 22 subjects (88.0%). The mental component score of the SF‐12 increased by 9.4 points (17.5%) to 63.0 ± 2.4 after 12 months of treatment, and the physical component score increased by 4.3 points (9.4%).

Sleep was assessed using the PSQ‐3, and patients reported improvement on all 3 questions. The mean score for trouble falling asleep decreased from 7.1 ± 0.5 at baseline to 1.8 ± 0.5 after 12 months of treatment (*P* < 0.001, paired *t*‐test), while the score for awakening during the night due to pain decreased from 7.1 ± 0.5 to 1.3 ± 0.3 (*P* < 0.001, paired *t*‐test), and the score for awakening in the morning due to pain decreased from 8.2 ± 0.4 to 2.2 ± 0.6 (*P* < 0.001, paired *t*‐test).

## Discussion

These results support the safety and efficacy of 10‐kHz SCS as a treatment option for CPSP. The safety results observed in this study demonstrated that the therapy does not introduce any unanticipated AEs. Study‐related AEs occurred in 7 subjects (20.6%), and no neurological deficits were observed.

In order to define a homogenous patient population, CPSP was defined by 3 criteria to produce a homogenous patient population while remaining independent of the location of pain: (1) pain caused or exacerbated by a surgical procedure; (2) pain limited to 1 or 2 dermatomes; and (3) pain likely to be neuropathic, as defined by a DN4 score of 4 or more.

Pain intensity showed rapid and continued improvement throughout the study period, and the responder rate compares favorably to previous studies of 10‐kHz SCS in other pain conditions, including the SENZA‐RCT randomized controlled trial, which reported response rates of 84.5% for back pain and 83.1% for leg pain, and other real‐world and prospective studies.^15^, ^16^, ^17^, ^28^, ^29^, ^30^, ^31^, ^32^, ^33^, ^34^, ^35^ The remission rate was 62.1% (18 subjects) after 1 year of treatment, which is very similar to the rate from SENZA‐RCT, which was about 60%.

To examine whether the population of patients with CPSP was indeed homogenous in pain etiology and response, the results from the entire cohort were compared to a subgroup analysis of the 10 patients with chronic postsurgical knee pain, and their reported pain scores before and after stimulation were very similar to the entire cohort. At 12 months, the responder rate in permanent implant patients (per‐protocol population) with postsurgical knee pain was 77.8%, and the remission rate was 66.7%, which was comparable to the overall rate of 62.1%, which suggests that 10‐kHz SCS is similarly effective in patients with CPSP, regardless of pain location.

Treating patients with 10‐kHz SCS also avoids some of the complications associated with alternative nonpharmacologic treatment options. Dorsal root ganglion stimulation presents risks similar to those of conventional SCS, but differences in the devices themselves and the implantation techniques may contribute to greater risks for dural puncture, neurologic injury, migration, and component damage associated with the former.^36^ As a paresthesia‐independent treatment modality, 10‐kHz SCS involves predictable anatomical epidural lead placement, reducing patient burden and procedure time.

Secondary endpoints, including patient function, mental health, well‐being, and sleep quality, also showed significant and sustained improvement, demonstrating broad benefits from this therapy. Affective descriptors in the SF‐MPQ‐2 questionnaire were significantly reduced after stimulation, demonstrating improvements in not just pain sensation but also the emotional component of pain.

It is recognized that greater patient attentiveness to pain and greater levels of catastrophizing can lead to higher pain intensity, greater emotional distress, greater levels of disability, and higher levels of healthcare use.^21^ The subjects in this study demonstrated high levels of pain catastrophizing at baseline, with a mean PCS score of 41.0, equivalent to the 93rd percentile of PCS scores in patients with clinical chronic pain.^20^ These scores decreased by 30.5 points to below the 30th percentile of PCS scores by the end of the study. We also found a 41.0% reduction in the PVAQ Attention to Pain subscale score and a 28.7% reduction in the Attention to Changes in Pain subscale score.

The mechanism of action of 10‐kHz SCS was demonstrated using animal experiments, namely by primarily reducing central sensitization through modulation of inhibitory interneurons in the superficial dorsal horn.^37^

This study was designed to prospectively evaluate 10‐kHz SCS as a treatment option for CPSP, as defined by the IASP and WHO, and these results support further investigation of this treatment to address unanswered questions. Subjects in this study were selected for presenting with probable neuropathic pain using DN4 scores, but only about 30% of patients with CPSP present with neuropathic pain features, depending on the type of surgery.^38^ It is unknown whether patients with nociceptive CPSP will respond to 10‐kHz SCS, so clinicians should reserve it for patients with refractory CPSP who have not responded to conservative medical treatments and make use of preventative measures.^39^

### Limitations

As an observational study, subjects were not randomized and there was no control group. The historical data presented is intended to provide context for the results and compensate somewhat for the lack of randomized controls. Moreover, the effect of selection bias was addressed by enrolling subjects in a variety of institutions, including a single‐physician practice, large multi‐physician practices, and a large research institution. Also, identical neuro‐assessments were not performed across all subjects. Although improvements in range of motion and sensation were reported, the results could not be compared between subjects since the neuro‐assessments were carried out only in the painful dermatomes in question. The study also did not collect information on opioid usage. The results need to be cautiously interpreted because sample size was small, and subjects had varied postsurgical peripheral nerve injury conditions.

## Conclusions

This is the first prospective study of 10‐kHz SCS in patients with CPSP, and the results support this therapy as a promising treatment option. The pain relief and responder rate in patients with permanent implants were comparable to 10‐kHz SCS studies in patients with chronic back and leg pain. Findings from the current study would encourage further studies into 10‐kHz SCS as a viable alternative for this unmet need in chronic pain.

## Conflicts of Interest

Dr. Gupta is on the scientific advisory board for Vertos Inc; consultant and speaker for Nevro Corp., Vertos Inc, and Foundation Fusion Solutions LLC; and received research grants from BIOTRINK, Sollis Therapeutics Inc, Allergan, Axsome Therapeutics Inc, SPR Therapeutics Inc, Nalu Medical Inc, and Vertiflex Inc. Dr. Scowcroft is a consultant for Nevro Corp. Dr. Kloster is a member of preceptorship faculty for Nuvectra, Vertos, Flowonix, and Vertiflex. Dr. Guirguis is a consultant for Boston Scientific and Avanos and received research grants from Abbott, Boston Scientific, and Neuros Medical. Dr. Carlson is a consultant for Nevro, Abbott, Saluda, Boston Scientific, Vertiflex, CornerLoc, and PillNurse. Dr. McJunkin is a consultant for Nevro Corp. and received research grants from Saluda Medical, Neuros Medical, Nevro, Abbott, and Stimgenics. Dr. Subbaroyan is employed by Nevro Corp. Drs. Chaiban and Israel have no conflicts of interest to disclose.

## Funding

The study was sponsored by Nevro Corp.

## Author Contributions

J.S. was responsible for conception and design planning, conduct, data analysis, and interpretation. M.G., J.S., D.K., M.G., J.C., T.M., G.C., and A.I. were involved in conduct, reporting, acquisition of data, and interpretation of data. All authors reviewed and approved the final manuscript.

## Supporting information


**Table S1.** Inclusion criteria.
**Table S2.** Exclusion criteria.
**Table S3.** Location of patient pain and surgeries.
**Figure S1.** Study design schematic.
**Figure S2.** Subject function improved after initiation of treatment.
