# Supplementary material for: 10‐kHz Spinal Cord Stimulation for Chronic Postsurgical Pain: Results From a 12‐Month Prospective, Multicenter Study
Source: Pain Pract. 2020 Jul 23;20(8):908–18. doi: 10.1111/papr.12929 (PMC7754504; doi:10.1111/papr.12929)

**Supplementary Table S1. Inclusion Criteria**

| 1. Have been diagnosed with chronic, focal, neuropathic pain following surgery of the trunk or the limb(s). 2. Average pain intensity (over a period of 7 days) of ≥5 out 10 cm on the Visual Analog Scale (VAS) in the primary area of pain at enrollment. 3. Have stable neurological status measured by motor, sensory and reflex function as determined by the investigator. 4. Be on stable pain medications, as determined by the Investigator, for at least 28 days prior to assessing pain intensity as described in inclusion criterion #2 and be willing to stay on those medications with no dose adjustments until activation of the permanently implanted SCS device. 5. Be 18 years of age or older at the time of enrollment. 6. Be an appropriate candidate for the surgical procedures required in this study based on the clinical judgment of the implanting physician. 7. Be able to read and understand English. 8. Be capable of subjective evaluation; patient must be able to describe and rate his/her pain levels. 9. Be willing and capable of giving informed consent. 10. Be willing and able to comply with study-related requirements, procedures, and visits. 11. Have adequate cognitive ability to use a patient programmer and recharger as determined by the Investigator. 12. Have a score of at least 4 out of 10 in Douleur Neuropathique 4 (DN4) questionnaire at the time of enrollment or eligibility criterion. |
| --- |

**Supplementary Table S2. Exclusion Criteria**

| 1. Have chronic back or trunk pain resulting from failed back surgery. 2. Have a medical condition or pain in other area(s), not intended to be treated with SCS, that could interfere with study procedures, accurate pain reporting, and/or confound evaluation of study endpoints, as determined by the Investigator (such as primary headache diagnosis or fibromyalgia). 3. Have a current diagnosis of a progressive neurological disease such as multiple sclerosis (MS), chronic inflammatory demyelinating polyneuropathy, rapidly progressive arachnoiditis, brain or spinal cord tumor, central deafferentation syndrome, acute herniating disc, severe spinal stenosis and brachial plexus avulsion as determined by the Investigator. 4. Have a current diagnosis or condition such as a coagulation disorder, bleeding diathesis, platelet dysfunction, progressive peripheral vascular disease or uncontrolled diabetes mellitus that presents excess risk for performing the procedure as determined clinically by the Investigator. 5. Have any prior experience with SCS. 6. Have objective evidence of epidural scarring and/or any signs or symptoms of myelopathy. 7. Be benefitting from an interventional procedure to treat their trunk or limb pain (Subjects should be enrolled at least 30 days from last benefit). 8. Have an existing drug pump and/or another active implantable device such as a pacemaker. 9. Have a condition currently requiring or likely to require the use of MRI of the trunk. 10. Have a condition currently requiring or likely to require the use of diathermy. 11. Have either a metastatic malignant neoplasm or untreated local malignant neoplasm. 12. Have a life expectancy of less than 1 year. 13. Have a local infection at the anticipated surgical entry site or an active systemic infection. 14. Be pregnant (participants of child-bearing potential that are sexually active must use a reliable form of birth control). 15. Have within 6 months of enrollment a significant untreated addiction to dependency producing medications, alcohol or illicit drugs. 16. Be concomitantly participating in another clinical study. 17. Be involved in an injury claim under current litigation. 18. Have an active or unsettled worker’s compensation claim. 19. Have evidence of an active disruptive psychological or psychiatric disorder or other known condition significant enough to impact perception of pain, compliance of intervention and/or ability to evaluate treatment outcome, as determined by a psychologist. |
| --- |

**Supplementary Table S3. Location of patient pain and surgeries**

| No. | Location of Pain | Surgery |
| --- | --- | --- |
| Upper Limbs | | |
| 1. | Right wrist/arm | Dorsal compartment release |
| 2. | Right wrist | Wrist arthroscopy |
| Trunk | | |
| 1. | Left chest wall; Left shoulder | Surgeries of left claviclosternum/scapula after motor vehicle accident |
| 2. | Abdomen | Gastric bypass, abdominoplasty, incisional hernias revised with mesh (several times) |
| 3. | Right rib cage; Right shoulder/arm | Pain post-video assisted thoracoscopy (VAT) |
| 4. | Right upper abdomen | Esophagotomy (2X) |
| 5. | Right ribs/Right back | VAT/Thoracotomy |
| 6. | Right ribs/Right shoulder | Thoracic rib fractures after fall from ladder (pins placed to fix ribs) |
| 7. | Mid-abdomen; Mid-chest | Partial gastrectomy, hernia repair |
| 8. | Left chest wall; back and neck | Double mastectomy |
| 9. | Abdomen | Cholecystectomy, oophorectomy, splenectomy, cyst removal |
| Lower Limbs | | |
| 1. | Right groin | Hernia repair |
| 2. | Left foot | Left foot hammer toe surgery |
| 3. | Foot/ankle | Surgery of the tibial region post-spider bite (Brown Recluse Spider) |
| 4. | Left knee | Total knee replacement |
| 5. | Left knee | Total knee replacement |
| 6. | Left knee | Total knee replacement |
| 7. | Left knee | Total knee replacement |
| 8. | Left knee | Total knee replacement |
| 9. | Left knee | Soft tissue release of the left knee |
| 10. | Left knee/Left foot | Ankle surgery post-Achilles tendon injury |
| 11. | Bilateral knees | Total knee replacement |
| 12. | Right knee | Knee arthroscopy, Total knee replacement |
| 13. | Right knee | Total knee replacement |
| 14. | Right ankle/Right foot | Ankle fusion |
| 15. | Right ankle/heel | Ankle fusion |
| 16. | Right ankle/foot | Bilateral ankle surgery |
| 17. | Right toe/foot | Multiple foot surgeries |
| 18. | Right foot/ankle | Surgeries to scrape (3X), pin and rod (2X) |
| 19. | Left foot | Lateral ankle stabilization, tarsal tunnel release, medial column surgery |
| 20. | Left foot | Foot surgeries post-fracture |
| 21. | Foot | Fasciotomy after deep vein thrombosis and compartment syndrome |
| 22. | Bilateral foot | Fasciotomy |
| 23. | Right ankle | Foot/toe arthroscopy |

**Supplementary Figure S1. Study design schematic.**


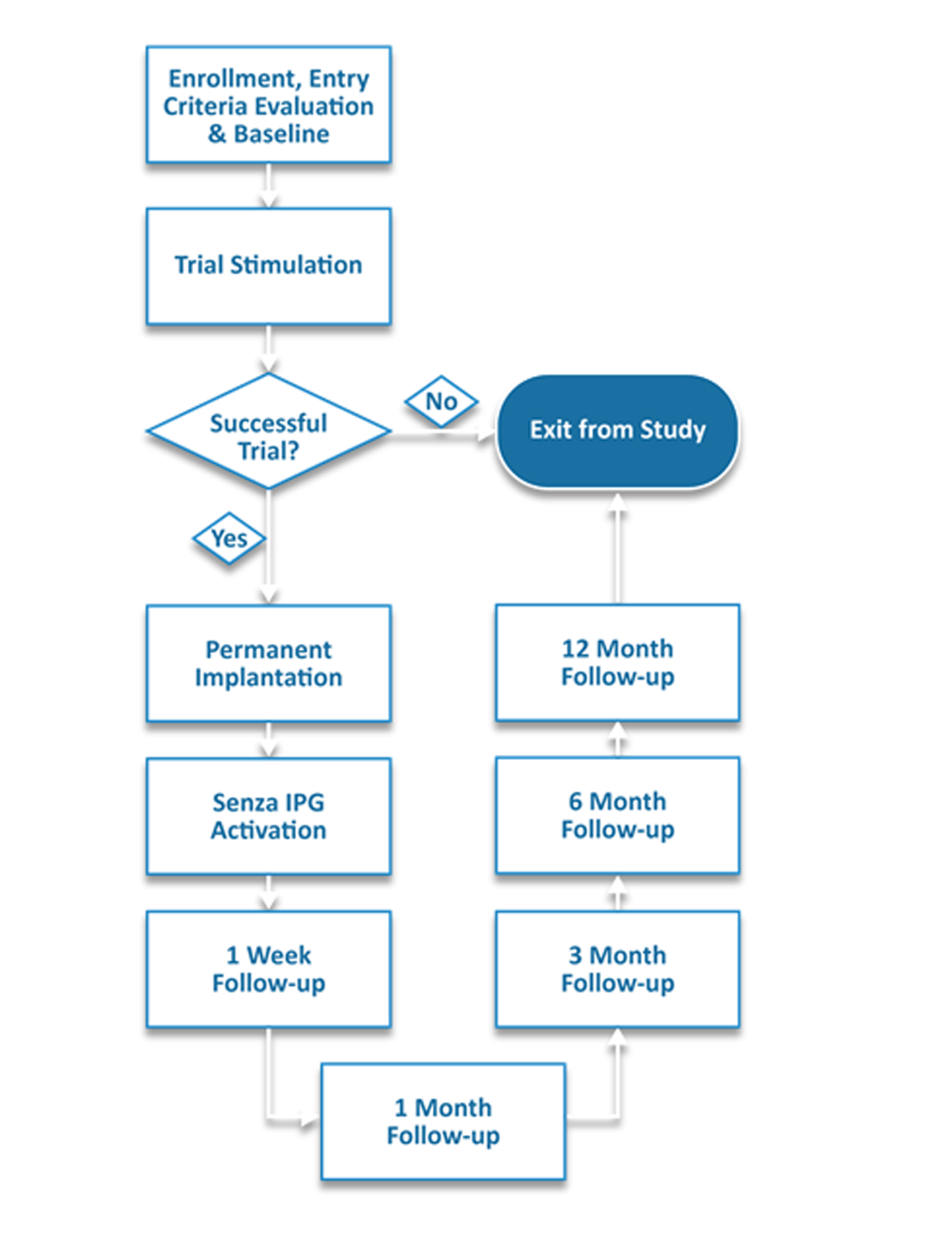


**Supplementary Figure S2. Subject function improved after initiation of treatment.** Mean PDI scores plotted at baseline and at 1, 3, 6, and 12 months after treatment initiation (left panel). Number of subjects exceeding MCID for PDI improvement at 1, 3, 6, and 12 months after treatment initiation (right panel).


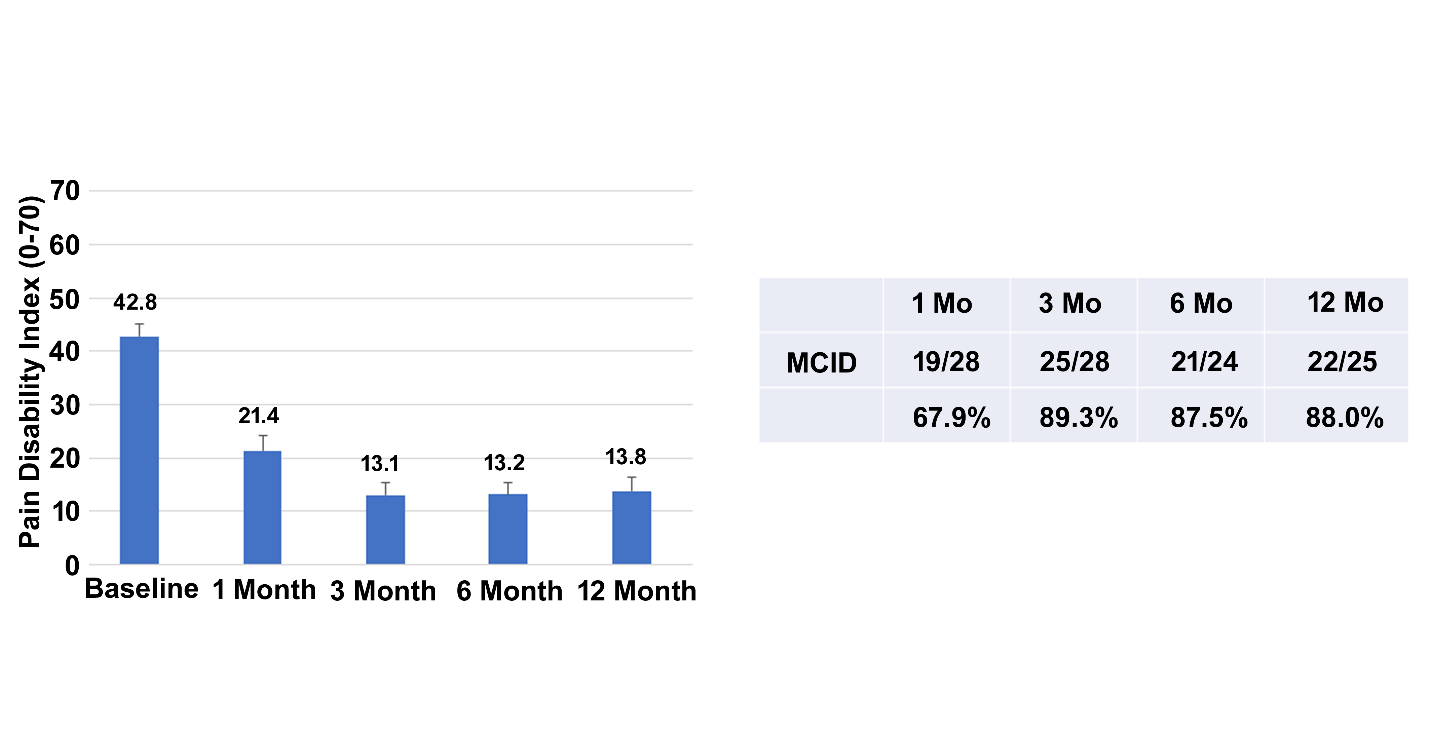

Supplement: Supplementary file 1 — Table S1. Inclusion criteria. Table S2. Exclusion criteria. Table S3. Location of patient pain and surgeries. Figure S1. Study design schematic. Figure S2. Subject function improved after initiation of treatment. [file PAPR-20-908-s001.docx]
